# Assessing the impact of the Physician Payments Sunshine Act on pharmaceutical companies’ payments to physicians

**DOI:** 10.1371/journal.pone.0306886

**Published:** 2024-08-13

**Authors:** Sherri Cheng, Wenjing Duan, Wenqi Zhou

**Affiliations:** 1 Department of Operations and Business Technology Management, Chaifetz School of Business, Saint Louis University, Saint Louis, Missouri, United States of America; 2 Department of Information Systems and Technology Management, School of Business, The George Washington University, Washington, D.C., United States of America; 3 Department of Accounting, Information Systems and Technology and Supply Chain, Palumbo Donahue School of Business, Duquesne University, Pittsburg, Pennsylvania, United States of America; Macquarie University, AUSTRALIA

## Abstract

Enacted in 2010 as part of the Affordable Care Act, the Physician Payments Sunshine Act (PPSA) mandates transparency in financial interactions between pharmaceutical companies and healthcare providers. This study investigates the PPSA’s effectiveness and its impact on industry payments to physicians. Utilizing ProPublica and Open Payments databases, a difference-in-difference analysis was conducted across ten states. Results reveal a significant reduction in pharmaceutical companies’ meal-related payments post-PPSA, impacting both the total payment amount and the number of unique physicians reached. Conversely, travel payments showed no significant impact in the primary analysis. However, subsequent analyses revealed nuanced reductions in the number of unique physicians reached, highlighting a more intricate relationship wherein pharmaceutical companies likely adjusted their financial interaction strategies with physicians differently across states. State-level variations in meals further underscore the complexity of PPSA’s influence. This pioneering research contributes valuable empirical evidence, addressing gaps in prior studies and emphasizing the ongoing need for policy assessment to guide industry-physician relationships.

## Introduction

The 2010 Physician Payments Sunshine Act (PPSA), part of the Affordable Care Act, mandates that pharmaceutical companies report their financial interactions with physicians and hospitals [1, 2]. Nationwide reporting has been in effect since August 2013, and data became available on the Centers for Medicare & Medicaid Services (CMS) Open Payments website in September 2014. The PPSA aims to increase transparency and monitor potential conflicts of interest [2, 3]. The intended impact of the PPSA includes curtailing inappropriate relationships between pharmaceutical companies’ marketing promotions and medical practitioners, empowering patients to make informed decisions, and facilitating unbiased clinical research conducted by physicians [2, 4–6].

In 2023, the PPSA marked its tenth anniversary; yet uncertainties persist regarding the policy’s effectiveness in achieving its intended objectives [7, 8]. Financial exchanges between pharmaceutical companies and physicians are among the most tangible and influential aspects illustrating the policy’s impact [9, 10]. These financial interactions have the potential to influence physicians’ prescribing behavior, raising concerns about a possible compromise in the quality of patient care [6, 10, 11]. Prior studies have examined similar state disclosure legislation’s impacts, mostly on physician prescription behavior, and have reached diverse conclusions [12–16]. The extension of such inquiries to examine the impact of the nationwide PPSA is considerably lacking, especially on the changes of overall payment. Recent research has examined the impact of the PPSA on pharmaceutical prescribing using only *post-PPSA* data [17–21]. These studies have identified a correlation between the PPSA and a reduction in physician prescription. It remains largely unexplored how the PPSA influences overall payment and its implications on pharmaceutical companies’ marketing strategies [7].

It is important to note that researchers have not yet established a *causal* link between the enactment of the PPSA and changes in industry payments. As pointed out by Richardson [2], assessing the ultimate empirical impact of the PPSA was challenging due to the limited availability of pre-PPSA data. This study is uniquely positioned with access to pre-PPSA payment data, aiming to address Richardson’s call [2] by providing more rigorous empirical evidence of the PPSA’s impact on industry payments to physicians.

We conducted a quasi-experimental study using eight years of official pharmaceutical payments that occurred before and after the enactment of the PPSA. Employing a difference-in-difference (DID) analysis, we assessed the PPSA’s impact on industry-physician exchanges. Our results indicate that the PPSA has indeed influenced payment interactions between companies and physicians. However, these effects varied notably between meals and travel payments. PPSA led to significant reductions in companies’ meal-related payments, affecting both the total payment made and the number of unique physicians reached. Nevertheless, the influence on travel payments appears to be less prominent, with variations observed across different states. This paper is among the pioneering efforts to examine the PPSA’s influence on the dynamics between key stakeholders, namely, manufacturers and physicians, by focusing on empirically identifying its *causal* impact on payment activities. With an estimated annual industry compliance cost of $180 million attached to PPSA regulation, as reported by CMS [22], revealing the PPSA’s effects on companies’ payments to physicians holds significant importance in assessing the policy’s efficacy, proposing ongoing enhancements, and justifying the substantial costs.

## Study data and methods

### Data

We compiled our physician payments data from two primary sources spanning the years 2012 to 2019. The data for the pre-PPSA period (2012–2013) was obtained from the proprietary ProPublica’s Dollars for Docs (PDFD) database. This database contains payment information derived from pharmaceutical companies’ disclosed payment reports, mandated by legal settlements with the federal government [21]. To promote low-cost data acquisition and data transparency, the data for the post-PPSA period (2014–2019) was collected from the open access Open Payments website (Open Payments: https://openpaymentsdata.cms.gov/). The Open Payment website is hosted by Centers for Medicare & Medicaid Services (CMS), which provides free and open access to payment data for the public. The original payment data from PDFD and Open Payments contains 50 U.S. states, Washington D.C., and four major U.S. territories.

The PDFD database is a substantial resource, comprising approximately 3.5 million official payment records from 17 major pharmaceutical companies. These records encompass nearly $4 billion in disclosed payments made between 2009 and 2013. This database provides comprehensive information about healthcare providers and the payments they have received from pharmaceutical and medical device companies for various activities, including consulting, education, meals, research, speaking engagements, and travel expenses. Since its inception, the PDFD data has been employed in scholarly works across both medical and data science fields [23–25]. The PDFD database allowed us to access payment information from the period before the PPSA enactment.

The validity and credibility of this study’s results rely on carefully matching these two data sources. To ensure the quality of the integrated final dataset, we made careful data choices and addressed several challenges. First, we opted to focus on two payment categories: meals and travel. These two categories were consistently defined by both data sources. It is worth noting that payments in the PDFD database were self-reported by individual companies without a standardized reporting template, and as a result, payment category definitions often lacked consistency across companies. To address this issue, we adopted the payment category definitions from Open Payments as the standard and subsequently examined the 155 payment categories in the PDFD database to identify potential matching categories. This process allowed us to establish a consistent framework for comparison and analysis between the two datasets, identifying the two consistently matched payment categories of meals and travel. Although some payment categories, such as speaking and consulting, may involve higher payments, they are not consistently defined and accessible from the existing database. Meanwhile, meals and travel payments are among the top three most frequent payment categories. According to the 2014 Open Payments data, among the 624,730 unique physicians across all 50 states in the U.S. who received payment, 93.63% of them received meals payments, 26.17% received education payments, and 11.56% received travel payments. Therefore, examining these two popular payment categories can provide valuable insights into the impact of PPSA on payments.

Second, to ensure comparability between the two databases, we aggregated all payment data by year. While the Open Payments database records payment occurrences by specific dates, the PDFD only provides annual aggregated data. Additionally, we adjusted all payment amounts to the monetary value of 2013 to account for inflation.

Third, we focused on four Big Pharma companies: AstraZeneca, Eli Lilly, Novartis, and Pfizer, because these companies had the most comprehensive and consistent data available throughout the entire study period. These four companies are also known for their extensive industry-physician exchanges and consistently ranked among the top 10 pharmaceutical companies by sales revenue [26, 27].

Fourth, due to data availability, we excluded eight states and three territories from the original dataset (55 states and territories). To compare payments before and after the PPSA enactment, the included state or territory must have annual payment data from all four selected Big Pharma for both payment categories throughout the entire study period, 2012–2019. Among the excluded 11 states and territories, Maine, Vermont, and West Virginia have prior disclosure laws. We excluded payment records prior to 2012, as many states had no payment records from most companies before that year.

In the end, we sorted 1,248,457 payment records from the PDFD database and obtained 786,231 records for two payment categories and four pharmaceutical companies, covering the years 2012 and 2013. Our post-PPSA industry payment data covers the period from 2014 to 2019 and is collected from the original data source, the Open Payments database, which encompasses all reported industry-physician payment activities from August 2013 to December 2022. We chose to use the 2014 to 2019 Open Payments data as our post-PPSA dataset for two main reasons. First, the Open Payments database had only four months of payment data available for 2013, so we utilized the PDFD payment data for that year. Second, we excluded data from 2020 and beyond, considering the inevitable impact of the COVID-19 outbreak on payments, to mitigate potential confounding factors and maintain a focused analysis of the policy impact. Our dataset from Open Payments comprises 9,313,680 payment records for two payment categories and four pharmaceutical companies, covering the years from 2014 to 2019.

In summary, our final dataset consisted of payment activities from four Big Pharma, focusing on two consistently defined payment categories: meals and travel. This dataset spans the years 2012 to 2019 and covers 44 states and territories. For the sake of brevity, we henceforth refer to these states and territories as the “44 states”.

### Method

Employing this dataset, we applied a well-established difference-in-difference (DID) method to examine the *causal* impact of the PPSA enactment on payment exchanges between companies and physicians through a quasi-experimental design [19, 28]. Researchers typically estimate the effect of a specific intervention or treatment (e.g., the passage of a law or the enactment of a policy) by comparing *changes* in outcomes over time between two comparable populations, one of which undergoes the treatment while the other does not. In our context, the PPSA represents the ‘treatment’, and it applies to the states that do not have prior disclosure mandates. We denote states with prior disclosure policies as disclosure states, and those without as non-disclosure states. Among the 44 states, we have three disclosure states, i.e., Massachusetts (MA), Minnesota (MN), and Washington D.C. (D.C.). They collectively constitute the population that has not undergone the treatment, serving as the candidates for the control group for DID analysis. However, we excluded D.C. from the formal DID analysis due to its unique status as the capital of the U.S., which makes it hard to find a comparable treatment state. The disclosure states in the DID analysis thus include MA and MN. The remaining 41 non-disclosure states are considered appropriate representations of populations that have undergone the treatment and serve as viable candidates for treatment group for DID analysis.

Following the literature [29], in our main DID model, we selected comparable non-disclosure states for MA and MN primarily based on their geographical proximity. States that share a border with these states are all included. For MA, the selected neighboring states are New Hampshire (NH), New York (NY), Connecticut (CT), and Rhode Island (RI). For MN, the neighboring states included are North Dakoda (ND), South Dakota (SD), Iowa (IA), and Wisconsin (WI). We also collected state-related data from the U.S. Bureau of Economic Analysis (BEA) and the American Community Survey and compiled lists of licensed physicians in each state from a Federation of State Medical Board report [30]. These data were instrumental in ensuring comparability between the disclosure states and the chosen non-disclosure states. We then applied equal weight (0.25) to each of the four neighboring states for each disclosure state and generated the industry payments data of comparable non-disclosure states [29]. The longitudinal data structure for our DID models comprises annual observations of payments both before and after the PPSA [31]. This structure serves as the foundation for our main DID model, which compares MA versus the four neighboring states and MN versus the four neighboring states.

We adopted two outcome variables to assess the policy impacts: *total payment* and the *number of unique physicians who received payments (physician count)*. *Total payment* represents the cumulative annual payments made by the four companies by state, year, and payment category. *Physician count* refers to the total count of unique physicians who received payments from the four companies by state, year, and payment category. Physicians may receive only one payment from one company or receive multiple payments from multiple companies, but each physician is counted only once in the physician count variable.

We also conducted three additional robustness tests for supplementary evidence. In the first robustness test (RT1), we only selected NY from the neighboring states as the matched non-disclosure treatment state for MA. Similarly, we chose WI as the treatment state for MN. In addition to the geographic proximity, these pairs were chosen based on comparable economic and socioeconomic status and patterns of inter-state commuters. Notably, among all neighboring states, NY and WI have the highest number of inter-state commuters to and from MA and MN, respectively. While each state possesses its unique historical, economic, and geographical characteristics, the substantial inter-state commuting between our control and treatment state pairs inherently holds the potential to foster cultural and social convergence. This, in turn, may gradually contribute to a more pronounced resemblance between these state pairs.

In the second robustness test set (RT2), we opted to exclude payment data from the year 2014. Since companies’ payment data on the Open Payments website only became accessible to the public after September 2014, it is plausible that the complete impact of the PPSA might not be adequately represented in the payments until 2015 and later, when the significance of payment transparency fully materialized. This choice led us to undertake two additional tests. The RT2A involved the subsequent re-conduction of our main DID model with the omission of the 2014 data. The RT2B replicated the first robustness test where only NY and WI served as the treatment states, and with the omission of the 2014 data.

For each DID model formulated above, we tested the parallel trend assumption and controlled for year-specific effects. An essential assumption of the DID model is that, in the absence of any policy intervention, the trends in the differences of outcome variables should remain consistent between disclosure and non-disclosure states. Thus, we formally estimated the DID regression model only after we affirmed that the model passed the parallel trend test. The detailed parallel trend test results are included in S1A Appendix.

Meanwhile, we recognized that payment exchanges can exhibit substantial variation across payment categories. For instance, payments categorized as meals are often frequent but relatively small in terms of amounts, whereas travel-related payments tend to be less frequent but of larger sums. Consequently, we conducted separate DID regression models for *meals* and *travel* payments. This approach allows us to potentially uncover the differential impact of the PPSA on payment exchanges based on their purpose.

## Study results

### Model-free descriptive analysis

Our analytical sample for the model-free descriptive analysis included all 44 states, four big pharmaceutical companies, two payment categories, across years from 2012 to 2019. The average annual total payment from meals and travel are around $27.06 million dollars and $13.11 million dollars respectively, and the average annual number of physicians receiving payments from meals and travel are 335,547 and 7,182 respectively. The total payments for meals are twice as high as the total travel payments, and approximately 46 times more physicians receive meal payments compared to travel payments. This disparity in both payment volume and physician involvement prompted our separate examination of these payment categories.

**Fig 1:**
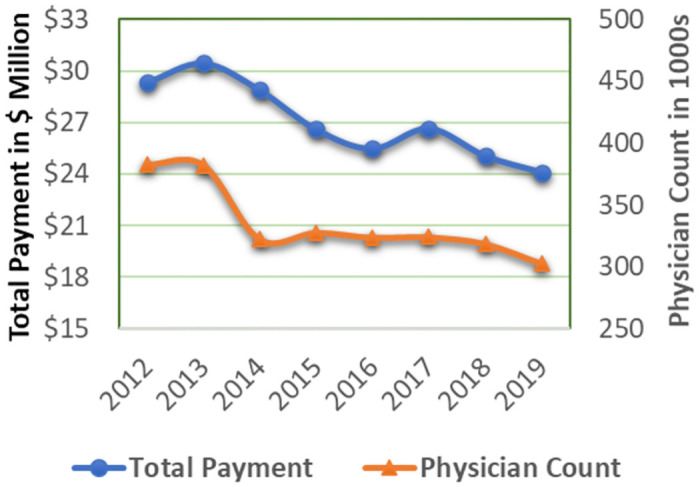
Historical payment exchange in meals category between pharmaceutical companies and physicians in 44 U.S. states, 2012–2019.

**Fig 2:**
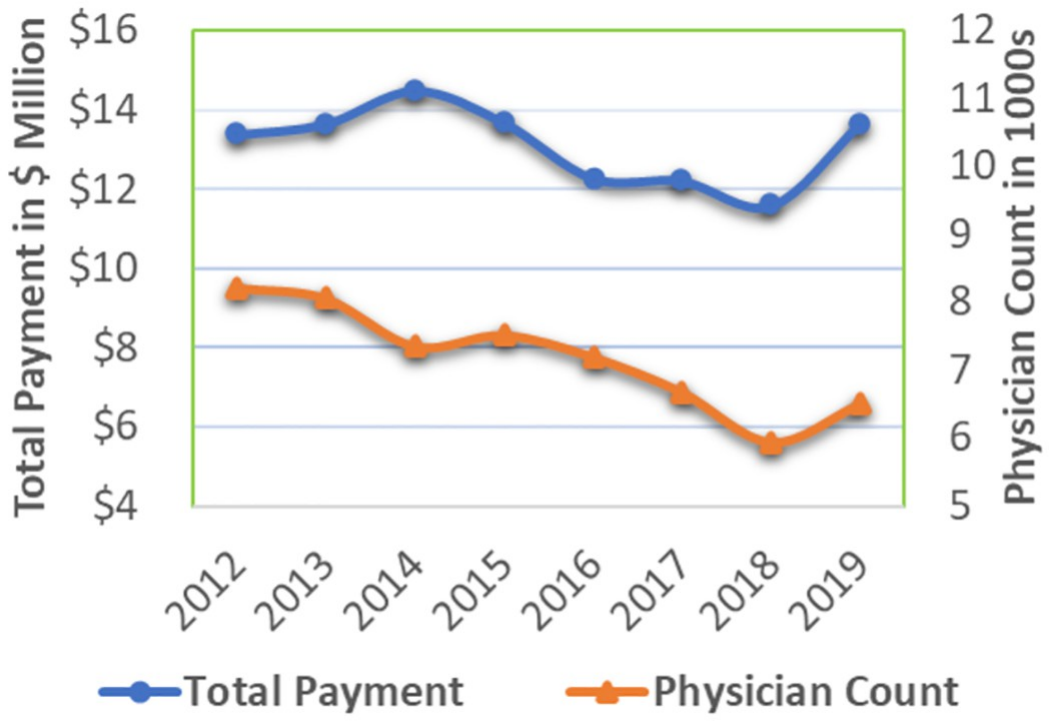
Historical payment exchange in travel category between pharmaceutical companies and physicians in 44 U.S. states, 2012–2019.

Fig 1 presents descriptive statistics for aggregated data from all 44 states, showcasing total payment and physician count for meals between 2012 and 2019. Fig 2 presents similar descriptive statistics for travel payments. The left Y-axis represents total payment in million-dollar units, while the right Y-axis represents physician count in thousands. We observe an overall declining trend in both meals and travel payments and physician count, indicating the potential impact of PPSA. In the case of meals, the reduction in payments and physician count began in 2014. For travel, the reduction in physician count emerged in 2014, while the decrease in total payment became evident in 2015. This echoes our robustness analyses in RT2A and RT2B by excluding payments from 2014.

**Fig 3:**
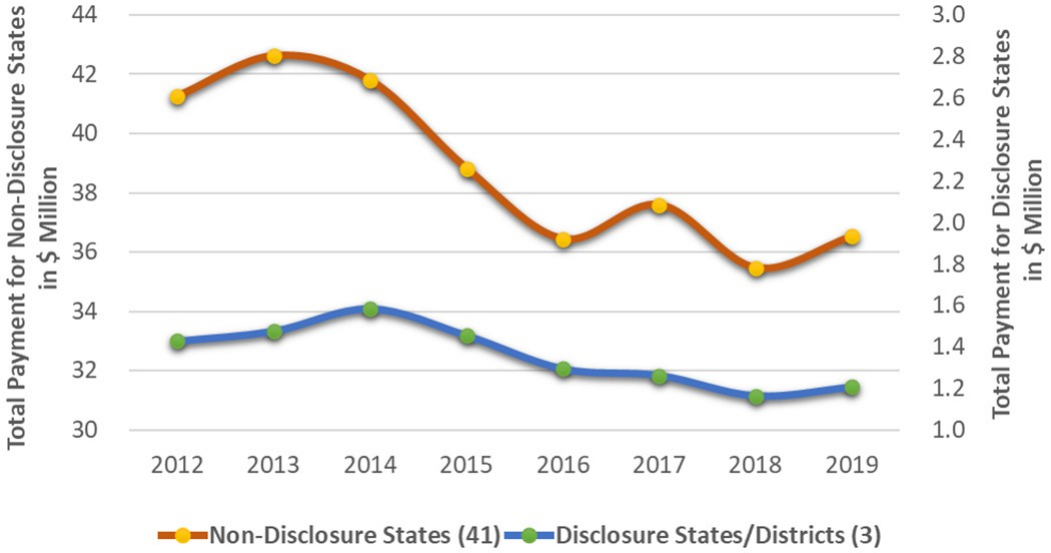
Historical payment exchange in meals and travel between pharmaceutical companies and physicians across the 44 U.S. states with prior disclosure laws compared to those without, 2012–2019.

We also aggregated payments for meals and travel and presented the trends for the total payment of the combined categories, while distinguishing between payments from disclosure states and payments from non-disclosure states (Fig 3). The left Y-axis represents total payment for both meals and travel in the 41 non-disclosure states, while the right Y-axis represents total payment for meals and travel in the three disclosure states. The units in Fig 3 are all denominated in million dollars. In general, we can observe a noticeable downward-sloping trend in the total payment for both types of states. Additionally, Fig 3 revealed that the observed increases in 2017 and 2019, as illustrated in Figs 1 and 2, predominantly occurred in the non-disclosure states. When comparing the total payment trends between disclosure and non-disclosure states, we observe milder changes in the former. While the descriptive analyses offer an initial visual link between the PPSA and the payments, it is crucial to conduct rigorous DID analyses to ascertain causal relationships and derive more robust inferences regarding the PPSA’s effect on total payment and physician count.

### Main DID results

Table 1 presents the estimation results of the main DID model, quantifying the changes in annual total payment and physician count between disclosure states (MA, MN) and neighboring non-disclosure states from before- to after-PPSA enactment (please refer to S1B Appendix for the details of quantifying the changes). In the meals category, overall, we observed a reduction in annual total payment and physician count in the non-disclosure states compared to the disclosure states. In the case of neighboring states to MA for meals payments, the annual physician count decreased by approximately 888 (*p* < 0.01) relative to MA. However, for annual meals payment, though reduced by over $113 thousand, it was not significant (*p* < 0.1). For MN’s neighboring state, physician meals payments experienced a reduction by over $47 thousand (*p* < 0.01), and the physician count decreased by 885 (*p* < 0.01) relative to MN. Surprisingly, for the travel payment category, we did not observe any statistically significant changes in total payment or physician count.

**Table 1:** Changes in annual total payment and physician count before and after the PPSA in the U.S., by payment categories, 2012–2019.

	Total Payment			Physician Count		
	Estimate	SE	AIC	Estimate	SE	AIC
**Meals** *(No*. *of OBS = 16)*						
Neighboring States vs. MA	-113085	57708	155.5	-887.71***	213.12	88.3
Neighboring States vs. MN	-47261***	10420	135.0	-885.00***	125.79	82.0
**Travel** *(No*. *of OBS = 16)*						
Neighboring States vs. MA	29019	60148	156.0	-13.29	14.54	56.1
Neighboring States vs. MN	-16036	15804	140.0	-16.75	8.75	50.0

Equal weight applied to each neighboring state.

*** *p* < 0.01.

### Robustness checks

Table 2 presents results for the robustness tests (RTs). RT1 estimates the PPSA’s impact using only NY and WI as the matching non-disclosure states, while RT2 shows the results when payments in 2014 were excluded from the regression estimation.

**Table 2:** Robustness checks results.

***Robustness test 1*: *2012–2013 vs*. *2014–2019 & one non-disclosure state vs*. *one disclosure state***						
	**Total Payment**			**Physician Count**		
	**Estimate**	**SE**	**AIC**	**Estimate**	**SE**	**AIC**
**Meals** *(No*. *of OBS = 16)*						
NY vs. MA	-496459**	184186	168.4	-6554.83***	590.32	100.5
WI vs. MN	-56937***	13700	138.2	-1056.50***	173.37	85.8
**Travel** *(No*. *of OBS = 16)*						
NY vs. MA	-37198	103663	162.5	-93.00**	34.32	66.4
WI vs. MN	-16827	15878	140.0	-15.50	7.71	48.5
***Robustness test 2A*: *2012–2013 vs*. *2015–2019 & four neighboring states vs*. *one disclosure state***						
	**Total Payment**			**Physician Count**		
	**Estimate**	**SE**	**AIC**	**Estimate**	**SE**	**AIC**
**Meals** *(No*. *of OBS = 14)*						
Neighboring states vs. MA	-137781**	40427.00	126.3	-931.63***	221.65	74.3
Neighboring states vs. MN	-51506***	7816.72	109.9	-919.65***	123.02	68.4
**Travel** *(No*. *of OBS = 14)*						
Neighboring states vs. MA	42120	61948.00	130.6	-17.40	13.98	46.7
Neighboring states vs. MN	-12712	16379.00	117.3	-15.70	9.58	42.9
***Robustness test 2B*: *2012–2013 vs*. *2015–2019 & one non-disclosure state vs*. *one disclosure state***						
	**Total Payment**			**Physician Count**		
	**Estimate**	**SE**	**AIC**	**Estimate**	**SE**	**AIC**
**Meals** *(No*. *of OBS = 14)*						
NY vs. MA	-581708**	110552	136.4	-6747.10***	532.75	83.1
WI vs. MN	-62555***	10196	112.6	-1102.60***	170.13	71.6
**Travel** *(No*. *of OBS = 14)*						
NY vs. MA	-74452	87812	134.0	-108.30***	22.38	51.4
WI vs. MN	-15034	17440	117.9	-15.20	8.64	41.8

Equal weight applied to each neighboring state.

** *p* < 0.05.

*** *p* < 0.01.

In RT1, we obtained overall consistent results to the main model in the meals payment category. Additionally, we observed a more statistically significant reduction in meal payments when comparing NY vs. MA (from *p < 0*.*1* in the main model to *p < 0*.*05* in RT1). Results in the travel category are also qualitatively similar for the measure of total payment, yet the physician count is shown to be reduced in the non-disclosure state NY, when compared to MA. On an annual basis, NY as a non-disclosure state has a decreased number of paid physicians by about 93 after the PPSA enactment. Overall, there is still a lack of strong evidence for the impact of PPSA on travel payments.

RT2 also yields fairly consistent results with the main model when excluding 2014 payments. Similar to RT1, both RT2A and RT2B also identify the statistically significant impacts of policy on the total meals payment for MA versus its counterparts. Comparing RT2A to the main model and RT2B to RT1 reveals that excluding 2014 data had little impact on the estimations of DID effects. RT2B shows results qualitatively similar to that of the main model, while aligning with RT1 regarding a significant yet minimal reduction in physician count in the travel category in NY versus MA.

Across all models, a coherent trend emerges, indicating that PPSA significantly reduces total payment and the physician count in the meals category. While robust tests highlight notable effects on reducing the physician count in the travel category in certain state pairs, an overarching sense of insignificance prevails with regard to the impact of PPSA on the two measured outcomes in the travel category as a whole.

## Discussion

It has been a decade since the launch of the PPSA. The PPSA sought to enhance transparency in financial transactions between healthcare providers and pharmaceutical manufacturers, aiming to instill greater accountability in the healthcare system. While some studies have explored the impact of state disclosure laws and demonstrated reduced payments, concerns and doubts exist about the effectiveness of PPSA as a national regulation [2]. As stated in [32], evaluating the effectiveness of healthcare policies requires independent evidence and should be conducted after a sufficient implementation period. Our study addresses this need by providing one of the earliest rigorous assessments of the PPSA’s impact on company-physician payment exchanges at an opportune time. Equipped with eight years of payment data, we are able to show the nontrivial impact of the nation-wide disclosure law on reducing payments, contributing to the debate about the efficacy of payment disclosure law.

In this study, we leverage a unique dataset compiled from two databases and apply a quasi-experimental design to assess the PPSA’s policy impact on the nature and scale of financial interactions between pharmaceutical companies and physicians. Our analysis provides robust evidence of the PPSA’s impact on meal-related payments, as demonstrated by our main DID model and corroborated through three robustness tests involving different control and treatment groups. These findings indicate a substantive policy effect in reducing meal-related payments to physicians, aligning with the PPSA’s objectives to promote transparency in financial relationships within the healthcare sector. This finding is significant, considering meals represent a common and perhaps more malleable avenue for industry-physician interactions.

In contrast, the impact of the PPSA on travel-related payments presents a more complex picture. While two out of four models indicated no statistically significant change in travel-related payments or the number of physicians accepting them, two robustness tests focusing on the pair of NY and MA suggest a likely reduction in the physician count receiving such payments. The mixed results prompt a contemplation of the policy’s scope and the multifaceted nature of industry-physician interactions. They also raise questions about the underlying factors that could account for the differential response in payment categories, such as the inherent characteristics of meal versus travel expenses or the varying degrees of scrutiny and reporting standards applied to them.

Our analysis also suggests that the observed effects in this study are attributable to the implementation of the PPSA itself, rather than just the public accessibility of its data. This assertion is supported by consistent results across our DID models, whether including or excluding payment data from 2014—a year when PPSA data was not fully available to the public.

While the payment reduction observed in our study cannot be conclusively linked to the mitigation of conflict of interest, the available anecdotal evidence is promising. Since the enactment of the PPSA, the Department of Justice (DOJ) has leveraged the Open Payment data in at least three significant lawsuits. In these cases, the data has served as critical evidence contributing to the successful prosecution of defendants for their misconduct. For instance, DOJ fined Medtronic over $9.2 million to settle allegations of improper payments to a South Dakota neurosurgeon in October 2020 [33]. The PPSA brings in the sunshine not only through transparency and heightened awareness of potential conflicts of interest but also through the value of the reported payments in law enforcement.

Our findings offer implications for policymakers. First, there is a vindication of the PPSA’s efficacy, suggesting that such transparency mandates can indeed reshape corporate conduct. Second, while the PPSA has made strides in certain areas, there remains an uneven landscape of impact. Future policy amendments may be necessary to address these variations and ensure the optimal impact of the PPSA is achieved. Third, our investigation also reveals a need to enhance public awareness of this policy and to promote the use of payment reports. There is a widespread consensus that the policy has not garnered significant attention from the general public [34, 35]. To address conflicts of interest in healthcare, it is imperative that patients—key beneficiaries of this policy—utilize the transparency provided by payment reporting to make more informed healthcare decisions. We recommend that CMS implement strategies to boost patient awareness regarding this policy and its associated payment reports. A promising model is found in California’s Assembly Bill 1278, which exemplifies an effective approach to promoting such awareness.

## Limitations and future research

This research leverages access to companies’ payment data in all states before the enactment of the national regulation law, PPSA. However, our analysis covers only four pharmaceutical companies and two payment categories to ensure rigorous validation and integration of data from PDFD and the Open Payments, prioritizing consistency and comprehensiveness. We observed from the Open Payments data that these four companies allocated a larger portion of payments to physicians in the meals and travel categories compared to the industry average. Furthermore, certain valuable categories like speaking, consulting, and research payments were excluded due to ambiguous definitions and/or insufficient data in the PDFD database. Additionally, PDFD only recorded annual payment exchange data. Future research could benefit from acquiring and studying more granular and comprehensive payment data.

The DID results effectively highlight the changes in company-physician payment exchanges resulting from the implementation of the PPSA. However, these results alone do not provide a comprehensive understanding of the dynamics underlying these changes. For example, when a significant change is identified, it remains uncertain whether it stems from reduced efforts by companies to engage with physicians, physicians’ reluctance to accept payments in response to the PPSA, or a combination of both factors. Meanwhile, with the limited data available, questions remain regarding whether the transparency induced by the PPSA is sufficient to deter inappropriate industry-physician exchanges, whether reductions in one payment category were offset by increases in other categories, and whether additional measures need to be taken by the policy. These issues are left for future research. Furthermore, the broader intended impacts of the PPSA on patient decision-making and the conduct of clinical research remain unexplored. To fully comprehend the policy’s complete impact, it is essential to delve into these aspects in future research.

## Conclusion

This research assessed the *causal* impact of PPSA enactment on financial exchanges between companies and physicians, revealing significant impacts on meal payments alongside nuanced impacts on travel payments. With a steadfast commitment to enhancing the reporting framework, amplifying the policy’s impact, and bolstering public awareness, we anticipate the policy’s full potential to become increasingly evident in the future.

## Supporting information

S1 Appendix **A.** Pre-trend analysis of the main DID model for total payment and physician counts. **B.** DID model specifications.
(PDF)
