# Supplementary material for: Assessing the impact of the Physician Payments Sunshine Act on pharmaceutical companies’ payments to physicians
Source: PLoS One. 2024 Aug 13;19(8):e0306886. doi: 10.1371/journal.pone.0306886 (PMC11321574; doi:10.1371/journal.pone.0306886)
Supplement: S1 Appendix — A. Pre-trend analysis of the main DID model for total payment and physician counts. B. DID model specifications. (PDF) [file pone.0306886.s001.pdf]

Supporting information

S1A Appendix. Pre-trend analysis of the main DID model for total payment and physician counts.

The following table records the parallel test results of the Main DID model.

Exhibit A1

Pre-Trend Analysis of the Main DID Model for Total Payment and Physician Count

Payment Type	Variable	DV: Total Payment			DV: Physician Count		
		Estimate	STD	*p*-Value	Estimate	STD	*p*-Value
**Meals**							
**Neighboring States vs. MA**	Treatment*Year2015	-330513	405344	0.4746	-2435.75	2980.38	0.4736
	Treatment*Year2016	-301646	405344	0.5108	-2545.25	2980.38	0.4559
	Treatment*Year2017	-248990	405344	0.5825	-1963.25	2980.38	0.5571
	Treatment*Year2018	-242115	405344	0.5924	-2236.25	2980.38	0.5075
	Treatment*Year2019	-196024	405344	0.6618	-2062	2980.38	0.5388
**Travel**							
**Neighboring States vs. MA**	Treatment*Year2015	274709	231766	0.3212	103.25	56.6859	0.1661
	Treatment*Year2016	216058	231766	0.42	81.75	56.6859	0.2449
	Treatment*Year2017	170185	231766	0.516	60	56.6859	0.3675
	Treatment*Year2018	172095	231766	0.5116	76.5	56.6859	0.27
	Treatment*Year2019	50351	231766	0.842	59.25	56.6859	0.3727
**Meals**							
**Neighboring States vs. MN**	Treatment*Year2015	-120202	154023	0.4921	-1798	2325.71	0.4958
	Treatment*Year2016	-114060	154023	0.5127	-1714	2325.71	0.5145
	Treatment*Year2017	-112464	154023	0.5181	-1577	2325.71	0.5463

	Treatment*Year2018	-113191	154023	0.5156	-1641.5	2325.71	0.5312
	Treatment*Year2019	-93453	154023	0.5869	-1370	2325.71	0.5972
**Travel**							
**Neighboring States vs. MN**	Treatment*Year2015	73022	51831	0.2536	44.5	24.0035	0.1608
	Treatment*Year2016	47365	51831	0.4282	40	24.0035	0.1942
	Treatment*Year2017	44431	51831	0.4543	27	24.0035	0.3425
	Treatment*Year2018	21894	51831	0.7012	14.5	24.0035	0.5884
	Treatment*Year2019	63425	51831	0.3084	22.5	24.0035	0.4177

SOURCE Authors’ analysis of 2012-2019 industry-physician payment exchanges.
NOTE The parallel tests measure changes in both total annual payment exchanges and annual counts of physicians receiving industry payments in states with disclosure statutes vs. their four neighboring states without disclosure statutes in the post-PPSA period. We calculate the DVs for each payment category by giving the neighboring states equal weight. The insignificant results show consistent support for the parallel trend assumption of the matched state pairs.

S1B Appendix. DID model specifications.

We estimated the following DID model equations, where *i* denotes state and *t* denotes year:

(1a) $\text{Total Payment}_{it}$

$$= \tau_t + \alpha \times \text{NoStatePolicy}_i \times \text{AfterPPSA}_t + \epsilon_{it}$$

(1b) $\text{Physician Counts}_{it}$

$$= \eta_t + \beta \times \text{NoStatePolicy}_i \times \text{AfterPPSA}_t + \zeta_{it}$$

Each equation was estimated in two payment categories, Meals and Travel, separately. AfterPPSA_t is a binary variable that we used to indicate whether year *t* was later than the PPSA implementation year. We set this variable to 1 for payments occurred after 2013, e.g., 2014–2019 (*after*) and 0 for 2012–2013 (*before*). NoStatePolicy_i is a binary variable that we used to indicate whether state *i* did not have PPSA-like state legislation before PPSA implementation. For example, since MN had a prior state disclosure policy; we set this binary variable to 0; we also set the binary variable for its matched counterparts, neighboring states and WI, to 1. The coefficients for the interaction term ($\text{NoStatePolicy}_i \times \text{AfterPPSA}_t$), α and β, are DID measures that capture the impact of the PPSA in the treatment state. τ_t and η_t, the time fixed effects, captured any time-invariant influences related to year *t*. The intended purpose of PPSA prompts us to expect that some α and β coefficients to be statistically significant and negative, implying that the nondisclosure state ($\text{NoStatePolicy}_i = 1$) has a payment reduction larger than the disclosure state.

Our main DID model, of which results are reported in Table 1, estimated the above two equations using payments data between 2012 and 2019 occurred in two pairs of treatment and control states, respectively: neighboring states vs. MA, neighboring states vs. MN. As explained in the manuscript, the DVs of neighboring states are calculated by applying equal weights, i.e., 0.25, on four individual neighboring states. The RT1 analysis estimated the above equations in a similar way but used two different pairs of control and treatment states separately: NY vs MA, WI vs. MN. The RT2 analysis replicated the main model and RT1 analysis by excluding payments data of 2014.
